# ABO and RhD blood groups as contributors to dyslipidaemia – a cross-sectional study

**DOI:** 10.1186/s12944-025-02444-6

**Published:** 2025-01-22

**Authors:** Malin Mickelsson, Kim Ekblom, Kristina Stefansson, Per Liv, Anders Själander, Ulf Näslund, Johan Hultdin

**Affiliations:** 1 Department of Medical Biosciences, Clinical Chemistry, Umeå University, Building 6M 2:Nd Floor, 901 85 Umeå, Sweden; 2 Department of Research and Development, Region Kronoberg, 351 12 Växjö, Sweden; 3 Department of Public Health and Clinical Medicine, Umeå University, 901 87 Umeå, Sweden

**Keywords:** ABO Blood-Group system, RhD blood group, Atherosclerosis, Dyslipidaemia

## Abstract

**Background:**

The ABO blood group system has shown an association with cardiovascular disease. The susceptibility to CVD is proposed to be partly mediated by dyslipidaemia in non-O individuals. Previous studies are scarce for the RhD blood group, but we recently showed that RhD − young individuals are associated with subclinical atherosclerosis. Hence, we sought to examine whether the ABO blood groups and RhD factor are associated with dyslipidaemia.

**Methods:**

All participants were part of the VIPVIZA study, including 3532 individuals with available plasma lipid levels. Lipids were assessed as total, LDL, HDL, remnant, non-HDL cholesterol and triglycerides. Information about ABO and RhD was retrieved by linking VIPVIZA with the SCANDAT-3 database, where 85% of VIPVIZA participants were registered.

**Results:**

For the ABO blood groups, no significant differences in lipid levels between non-O and O individuals were seen. In 40-year-old males, RhD − individuals compared to RhD + had higher levels of non-HDL cholesterol, LDL cholesterol, and remnant cholesterol, with ratios of geometric means of 1.21 (CI95% 1.03; 1.43), 1.20 (1.02; 1.41) and 1.38 (1.00; 1.92), respectively. No differences in lipid levels depending on the RhD blood group were seen in women or the older age groups.

**Conclusion:**

Our study indicates that younger RhD − men have increased non-HDL, LDL, and remnant cholesterol levels. Thus, the RhD blood group, but not ABO, seems to be associated with dyslipidaemia and may act as a future possible risk marker of cardiovascular disease.

**Graphical Abstract:**

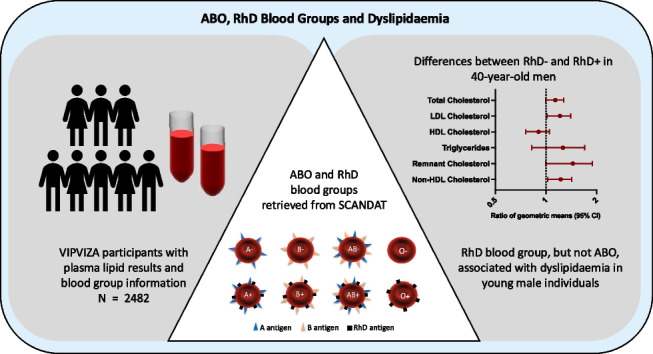

**Supplementary Information:**

The online version contains supplementary material available at 10.1186/s12944-025-02444-6.

## Introduction

Cardiovascular diseases (CVDs) are the leading cause of mortality and morbidity worldwide [1]. Several modifi and causal risk factors, such as hypertension, apoB-containing lipoproteins, smoking, diabetes, and obesity, have been presented. These five risk factors have been shown to represent a global population attributable risk of 57.2% in women and 52.5% in men [2].

However, a significant proportion of individuals have none of the traditional risk factors [3], and to improve the precision of risk assessment and prevention guidelines, new risk markers are crucial to find.

Blood groups are inherited constant markers and can be categorised based on different systems. The classification into different blood groups is based on the presence of antigens on the erythrocyte’s cell surface, whereby ABO and RhD are the most frequently applied in clinical practice. For ABO, there are four possible blood types: A, B, AB, and O, corresponding to if A, B, both A and B, or none of the antigens are present. For RhD, the presence or absence of the RhD protein determines the blood group, resulting in RhD + or RhD − as possible blood types [4].

Several associations between ABO phenotypes and increased susceptibility to disease have been reported in extensive studies and systematic reviews [5–7]. The non-O blood group has shown an increased risk for venous and arterial thrombotic vascular disease [8, 9], coronary atherosclerosis, and peripheral vascular disease [10, 11]. It has been proposed that this susceptibility in non-O blood groups is mediated partly by dyslipidaemia [12–15]. However, there is conflicting evidence [16, 17], and other mechanisms in the pathogenesis of atherosclerosis, such as coagulation and inflammation pathways, have also been presented [17–19]. Meanwhile, few studies are available on the RhD blood group in general. Still, the RhD factor was recently associated with subclinical atherosclerosis in younger individuals [20], and associations with dyslipidaemia have previously been presented [21].

In this study, we sought to investigate whether the ABO blood groups and RhD factor are associated with dyslipidaemia. We explored this by examining healthy individuals with available plasma lipid levels and retrieved their blood group from the SCANDAT database.

## Patients and methods

### Study population and covariates

All individuals were participants of the VIPVIZA trial (ClinicalTrials.gov NCT01849575), a pragmatic randomised controlled trial integrated into the Västerbotten intervention programme (VIP) with a previously available study protocol [22]. VIP is a population-based cardiovascular prevention programme offered to all residents in Västerbotten County the year they turn 40, 50, or 60 since the 1990s within primary care [23, 24]. The programme includes health counselling and risk factor screening, such as measurement of systolic and diastolic blood pressure, waist circumference, body mass index (BMI), and blood samples for analysis of lipid parameters and oral glucose tolerance tests. Participants also complete extensive questionnaires covering socioeconomic and psychosocial conditions.

VIP participants were further invited to the VIPVIZA trial at the individual interview within VIP if they met the following criteria: For the 60-year group, age alone was the inclusion criterion, i.e. all 60-year-olds were invited. For the 50-year group, inclusion required one or more of the following: hypertension, LDL cholesterol > 4.5 mmol/L, waist circumference > 102 cm for men and > 88 cm for women, smoking, diabetes, or a first-degree relative with CVD disease before 60 years of age. For the 40-year group, the criterion was a first-degree relative with a cardiovascular disease history before 60. In total, 3532 were included from 2013 to 2016, previously described in detail [22].

**Fig. 1:**
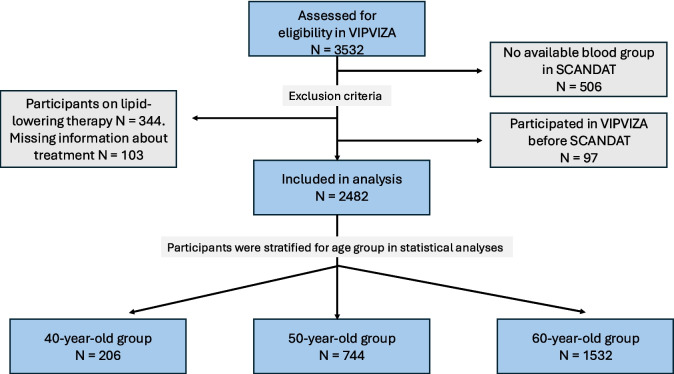
Flowchart visualising exclusion criteria and the number of included participants in different age groups

In this study, participants with self-reported use of lipid-lowering therapy or missing information about lipid-lowering therapy were excluded to avoid interference on lipid variables. This exclusion accounted for 447 individuals, where 344 were excluded due to statin treatment and 103 due to missing information about treatment (see Fig. 1). The proportion of subjects in different age groups using statins was 5.9%, 10.7%, and 18.9% in the 40-, 50- and 60-year-old groups, respectively.

Additionally, 3, 25 and 16 individuals in the 40, 50 and 60-year-old groups, respectively, had missing values for LDL cholesterol and consequently also remnant cholesterol. This is because the Friedewald formula for calculating LDL is inaccurate with triglyceride levels > 4.0 [25]. These individuals were exluded in the statistical analyses regarding LDL and remnant cholesterol, but not for anlysis of the other lipid parameters.

Smoking was categorised as smoking daily or not (including former smokers and never smokers) according to the self-reported data in the questionnaire. Diabetes was defined as self-reported and/or according to WHO guidelines, having fasting plasma glucose ≥ 7.0 mmol/L, and/or an oral glucose tolerance test with a 2-h post-load plasma glucose ≥ 12.2 mmol/L as capillary plasma was used [26].

### ABO and RhD blood groups

We retrieved information about ABO and RhD blood groups by linking the Swedish-Danish database (SCANDAT-3) to VIPVIZA. The SCANDAT database includes records of blood groups in individuals who have had any blood group testing done, for example, during routine controls in pregnancy, when receiving blood transfusions, or donating blood [27]. To avoid interference from the VIPVIZA trial on blood typing indication, 97 individuals who participated in VIPVIZA before SCANDAT registrations were excluded. Additionally, 506 VIPVIZA individuals had missing information about their blood group in SCANDAT (see Fig. 1). Thus, we retrived blood group information from SCANDAT in 2929 participants, corresponding to approximately 83% of all VIPVIZA participants. Differences between included and excluded participants have previously been described in a drop-out analysis [20].

### Sample collection and biochemical analysis

Venous blood samples were obtained after overnight fasting during the original VIP survey. Plasma for analysis of lipid parameters was analysed using standard hospital assays at the Department of Laboratory Medicine, Clinical Chemistry, University Hospital Umeå, accredited according to ISO 15189:2012 and Swedac accreditation no 1397.

Total cholesterol, HDL cholesterol and triglycerides were measured with enzymatic colourimetric methods. LDL-cholesterol was calculated using the Friedewald formula [25]. Non-HDL cholesterol and remnant cholesterol were calculated using the following equations: Non-HDL cholesterol = total cholesterol − HDL cholesterol and remnant cholesterol = total cholesterol − HDL cholesterol – LDL cholesterol.

### Statistical methods

Baseline characteristics for different blood groups were presented as numbers and percentages for categorical variables and as medians (25th to 75th percentiles) for continuous variables. The chi-square test was used to test whether the proportions of RhD − /RhD + individuals were different across the age groups as well as in the ABO blood groups.

Due to positively skewed distributions, all lipid variables (total, LDL, HDL, non-HDL, remnant cholesterol, and triglycerides) were logarithmically transformed using base 10. The associations between different lipid levels and blood groups (non-O vs. O and RhD + vs. RhD −) were assessed by t-tests, stratified by age group and sex. Differences in lipid variables between blood groups on the logarithmic scale, including the 95% confidence intervals (CI 95%), were retransformed using the base-10 exponential function to the original scale and interpreted as a ratio of geometric means. The ratio of geometric means (and CI 95%) with a value > 1 implies that the mean lipid value was higher among RhD** − **compared to RhD + .

Additionally, a subgroup analysis was performed on participants reporting CVD heredity since blood group is a hereditary factor. Heredity was defined as having a first-degree relative with CVD before 60 years of age. This corresponded to all individuals in the 40-year-old group since it was an inclusion criterion for VIPVIZA. For the 50- and 60-year-old groups, we retrieved information about heredity from the original VIP questionnaire, 10 or 20 years prior, when these participants were also 40.

All calculations were performed with SPSS version 28 (IBM corporation. New York, NY, USA). Figures were created in GraphPad Prism 10.3.0 (461) Macintosh Version by Software MacKiev (Boston, MA, USA).

## Results

The A, B, AB, and O distribution was 43.4%, 12.6%, 6.2%, and 37.8%, respectively. Thus, 62.2% of participants were classified as having a non-O blood group. Baseline characteristics for the ABO blood group, including the distribution of lipid levels stratified for sex and age group, are presented in Table 1. In total, 62.2% were classified as having a non-O blood group (62.4% among women and 62.0% among men) and 85.4% were classified as RhD + (86.0% among women and 84.6% among men). The baseline characteristics for RhD stratified for age group and sex are presented in Table 2. There were no differences in the frequency of RhD + individuals between the age groups in both sexes, *p* = 0.82 and *p* = 0.90 for men and women, respectively. Nor were there any differences in the proportions of non-O and O between the age groups, *p* = 0.99 for men and *p* = 0.30 for women.

**Table 1:** Baseline descriptives for the ABO blood groups stratified for sex

	**Men**		**Women**	
	**O**	**Non-O**	**O**	**Non-O**
All subjects N (%)	380 (38.0)	619 (62.0)	557 (37.6)	926 (62.4)
40-year old group N (%)^a^	29 (38.2)	47 (61.8)	57 (43.8)	73 (56.2)
50-year old group N (%)^a^	112 (37.7)	185 (62.3)	166 (37.1)	281 (62.9)
60-year old group N (%)^a^)	239 (38.2)	387 (61.8)	334 (36.9)	572 (63.1)
Hypertension N (%) all	206 (54.2)	338 (54.6)	231 (41.5)	420 (45.4)
40 y	10 (34.5)	18 (38.3)	12 (21.1)	11 (15.1)
50 y	60 (53.6)	102 (55.1)	80 (48.2)	124 (44.1)
60 y	136 (56.9)	218 (56.3)	139 (41.6)	285 (49.8)
Diabetes N (%) all	26 (6.8)	34 (5.5)	22 (3.9)	41 (4.4)
40 y	0	1 (2.1)	3 (5.3)	2 (2.7)
50 y	7 (6.3)	10 (5.4)	5 (3.0)	14 (5.0)
60 y	19 (7.9)	23 (5.9)	14 (4.2)	25 (4.4)
Smoking N (%) all	49 (12.9)	86 (13.9)	66 (11.8)	125 (13.5)
40 y	6 (20.7)	8 (17.0)	3 (5.3)	4 (5.5)
50 y	17 (15.2)	28 (15.1)	23 (13.9)	42 (14.9)
60 y	26 (10.9)	50 (12.9)	40 (12.0)	79 (13.8)
Lipid variables				
Total-Cholesterol (mmol/L) all	5.60 (4.90; 6.20)	5.70 (5.00; 6.40)	5.70 (5.10; 6.30)	5.70 (5.00; 6.40)
40 y	4.80 (4.60; 5.70)	5.20 (4.70; 6.10)	4.70 (4.30; 5.30)	4.65 (4.30; 5.30)
50 y	5.70 (4.93; 6.38)	5.80 (5.00; 6.60)	5.60 (5.00; 6.10)	5.50 (4.90; 6.10)
60 y	5.60 (4.90; 6.30)	5.70 (5.00; 6.30)	5.90 (5.30; 6.40)	5.90 (5.30; 6.60)
LDL cholesterol (mmol/L) all	3.60 (3.00; 4.30)	3.70 (3.10; 4.40)	3.60 (3.00; 4.20)	3.60 (3.00; 4.20)
40 y	3.20 (2.60; 3.65)	3.30 (2.85; 3.95)	2.80 (2.50; 3.45)	2.90 (2.40; 3.40)
50 y	3.70 (3.20; 4.30)	3.80 (3.10; 4.50)	3.60 (2.90; 4.15)	3.50 (3.00; 4.00)
60 y	3.70 (3.00; 4.39)	3.70 (3.10; 4.38)	3.70 (3.10; 4.30)	3.70 (3.20; 4.40)
HDL cholesterol (mmol/L) all	1.20 (1.00; 1.40)	1.20 (1.01; 1.42)	1.49 (1.25; 1.83)	1.47 (1.22; 1.77)
40 y	1.15 (0.97; 1.39)	1.23 (0.92; 1.40)	1.33 (1.20; 1.62)	1.29 (1.13; 1.57)
50 y	1.12 (0.97; 1.26)	1.10 (0.94; 1.40)	1.48 (1.20; 1.79)	1.37 (1.18; 1.67)
60 y	1.21 (1.03; 1.45)	1.23 (1.05; 1.45)	1.50 (1.31; 1.86)	1.54 (1.28; 1.84)
Triglycerides (mmol/L) all	1.35 (1.00; 1.90)	1.41 (0.99; 1.92)	1.10 (0.85; 1.50)	1.13 (0.84; 1.57)
40 y	1.17 (0.93; 1.93)	1.29 (0.92; 1.96)	0.83 (0.69; 1.18)	0.88 (0.66; 1.19)
50 y	1.50 (1.06; 2.11)	1.64 (1.03; 2.34)	1.03 (0.86; 1.50	1.13 (0.83; 1.61)
60 y	1.29 (1.00; 1.80)	1.37 (0.96; 1.82)	1.14 (0.90; 1.55)	1.18 (0.89; 1.60)
Non-HDL cholesterol (mmol/L) all	4.41 (3.60; 5.07)	4.46 (3.67; 5.17)	4.10 (3.45; 4.81)	4.18 (3.54; 4.90)
40 y	3.60 (3.20; 4.72)	4.21 (3.42; 5.00)	3.26 (2.96; 3.96)	3.32 (2.77; 4.00)
50 y	4.65 (3.84; 5.26)	4.56 (3.71; 5.51)	4.09 (3.42; 4.76)	4.02 (3.54; 4.70)
60 y	4.32 (3.60; 5.03)	4.42 (3.70; 5.05)	4.25 (3.70; 4.94)	4.31 (3.66; 5.06)
Remnant cholesterol (mmol/L) all	0.60 (0.47; 0.85)	0.63 (0.44; 0.85)	0.50 (0.39; 0.68)	0.51 (0.39; 0.70)
40 y	0.52 (0.39; 0.85)	0.56 (0.39; 0.82)	0.40 (0.31; 0.51)	0.40 (0.30; 0.50)
50 y	0.65 (0.48; 0.92)	0.68 (0.48; 0.95)	0.50 (0.39; 0.67)	0.52 (0.39; 0.74)
60 y	0.60 (0.47;0.81)	0.61 (0.44; 0.81)	0.52 (0.40; 0.71)	0.53 (0.40; 0.70)

Continuous variables presented as medians (25th to 75th percentiles) and categorial variables as numbers (percent)

^a^Percentages of ABO blood groups within strata are presented

**Table 2:** Baseline descriptives for the RhD blood groups stratified for sex

	**Men**		**Women**	
	**RhD + **	**RhD-**	**RhD + **	**RhD-**
All subjects N (%)	845 (84.6)	154 (15.4)	1275 (86.0)	208 (14.0)
40-year old group N (%)^a^	63 (82.9)	13 (17.1)	112 (86.2)	18 (13.8)
50-year old group N (%)^a^	254 (85.5)	43 (14.5)	387 (86.6)	60 (13.4)
60-year old group N (%)^a^	528 (84.5)	98 (15.7)	776 (85.7)	130 (14.3)
Hypertension N (%) all	459 (54.3)	85 (55.2)	546 (42.8)	105 (50.5)
40 y	25 (39.7)	3 (23.1)	16 (14.3)	7 (38.9)
50 y	141 (55.5)	21 (48.8)	176 (45.5)	28 (46.7)
60 y	293 (55.5)	61 (62.2)	354 (45.6)	70 (53.8)
Diabetes N (%) all	48 (5.7)	12 (7.8)	53 (4.2)	10 (4.8)
40 y	0	1 (7.7)	3 (2.7)	2 (11.1)
50 y	14 (5.5)	3 (7.0)	16 (4.1)	3 (5.0)
60 y	34 (6.4)	8 (8.2)	34 (4.4)	5 (3.8)
Smoking N (%) all	115 (13.6)	20 (13.0)	154 (12.1)	37 (17.8)
40 y	10 (15.9)	4 (30.8)	4 (3.6)	3 (16.7)
50 y	38 (15.0)	7 (16.3)	51 (13.2)	14 (23.3)
60 y	67 (12.7)	9 (9.2)	99 (12.8)	20 (15.4)
Lipid variables				
Total cholesterol (mmol/L) all	5.60 (4.90; 6.30)	5.70 (5.00; 6.40)	5.70 (5.10; 6.40)	5.70 (5.00; 6.50)
40 y	4.90 (4.50; 5.90)	5.60 (4.95; 6.25)	4.60 (4.30; 5.30)	4.75 (4.38; 5.48)
50 y	5.70 (4.98; 6.50)	6.00 (5.00; 6.50)	5.50 (4.90; 6.10)	5.40 (5.10; 6.25)
60 y	5.70 (5.00; 6.30)	5.60 (5.00; 6.30)	5.90 (5.30; 6.60)	5.90 (5.18; 6.60)
LDL cholesterol (mmol/L) all	3.70 (3.00; 4.40)	3.75 (3.13; 4.28)	3.60 (3.00; 4.20)	3.60 (3.00; 4.30)
40 y	3.20 (2.65; 3.70)	3.70 (3.15; 4.45)	2.90 (2.40; 3.40)	2.80 (2.55; 3.53)
50 y	3.70 (3.10; 4.40)	4.00 (3.38; 4.33)	3.50 (2.90; 4.00)	3.55 (3.10; 4.28)
60 y	3.70 (3.10; 4.40)	3.70 (3.10; 4.15)	3.70 (3.20; 4.30)	3.70; 3.10; 4.48)
HDL cholesterol (mmol/L) all	1.20 (1.01; 1.41)	1.17 (1.00; 1.39)	1.49 (1.23; 1.80)	1.46 (1.22; 1.75)
40 y	1.23 (0.99; 1.40)	1.01 (0.84; 1.26)	1.30 (1.13; 1.57)	1.41 (1.24; 1.63)
50 y	1.11 (0.95; 1.33)	1.10 (0.93; 1.30)	1.40 (1.19; 1.71)	1.40 (1.18; 1.60)
60 y	1.23 (1.04; 1.46)	1.20 (1.05; 1.40)	1.54 (1.30; 1.86)	1.49 (1.23; 1.80)
Triglycerides (mmol/L) all	1.37 (0.99; 1.88)	1.51 (1.06; 2.10)	1.11 (0.85; 1.54)	1.13 (0.83; 1.55)
40 y	1.17 (0.92; 1.71)	1.62 (0.92; 2.64)	0.88 (0.67; 1.20)	0.78 (0.63; 0.97)
50 y	1.52 (1.03; 2.27)	1.74 (1.17; 2.35)	1.10 (0.86; 1.54)	1.09 (0.80; 1.58)
60 y	1.32 (0.96; 1.80)	1.43 (1.02; 1.90)	1.16 (0.88; 1.57)	1.20 (0.90; 1.65)
Non-HDL cholesterol (mmol/L) all	4.42 (3.61; 5.13)	4.50 (3.75; 5.13)	4.14 (3.53; 4.82)	4.16 (3.42; 5.00)
40 y	3.68 (3.17; 4.66)	4.67 (3.82; 5.35)	3.31 (2.86; 4.00)	3.14 (2.88; 4.07)
50 y	4.54 (3.70; 5.34)	4. 70 (3.97; 5.38)	4.04 (3.50; 4.70)	4.04 (3.58; 4.95)
60 y	4.42 (3.65; 5.06)	4.40 (3.69; 4.93)	4.30 (3.68; 5.00)	4.30 (3.60; 5.17)
Remnant cholesterol (mmol/L) all	0.61 (0.45; 0.83)	0.66 (0.50; 0.93)	0.50 (0.39; 0.70)	0.50 (0.38; 0.70)
40 y	0.53 (0.38; 0.74)	0.69 (0.45; 1.20)	0.41 (0.30; 0.53)	0.33 (0.28; 0.33)
50 y	0.67 (0.48; 0.92)	0.72 (0.50; 0.99)	0.51 (0.40; 0.70)	0.50 (0.36; 0.74)
60 y	0.60 (0.44; 0.80)	0.65 (0.50; 0.87)	0.52 (0.40; 0.70)	0.53 (0.40; 0.74)

Continuous variables presented as medians (25th to 75th percentiles) and categorial variables as numbers (percent)

^a^Percentages of RhD blood groups within strata are presented

**Fig. 2:**
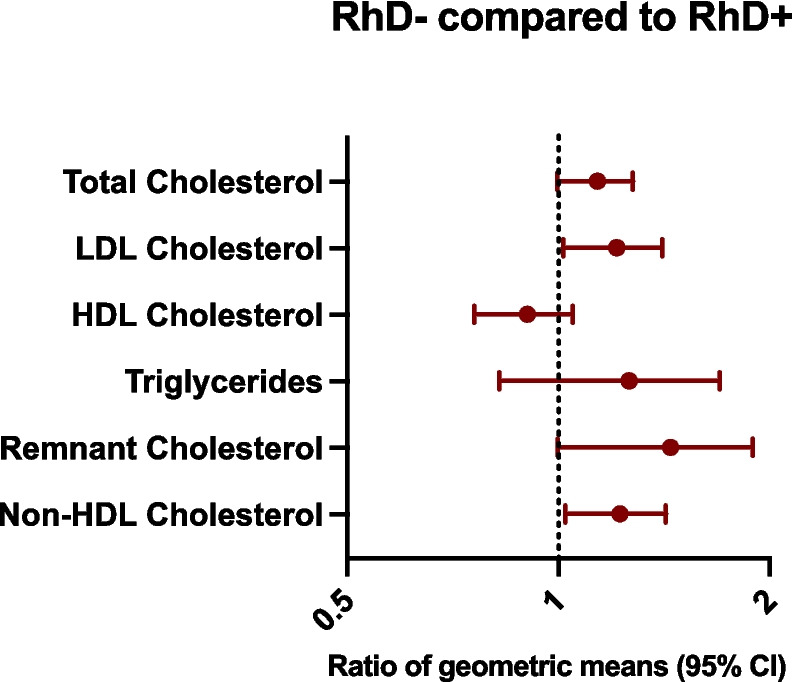
Differences in lipid levels in 40-year-old male RhD − compared to RhD + individuals

**Fig. 3:**
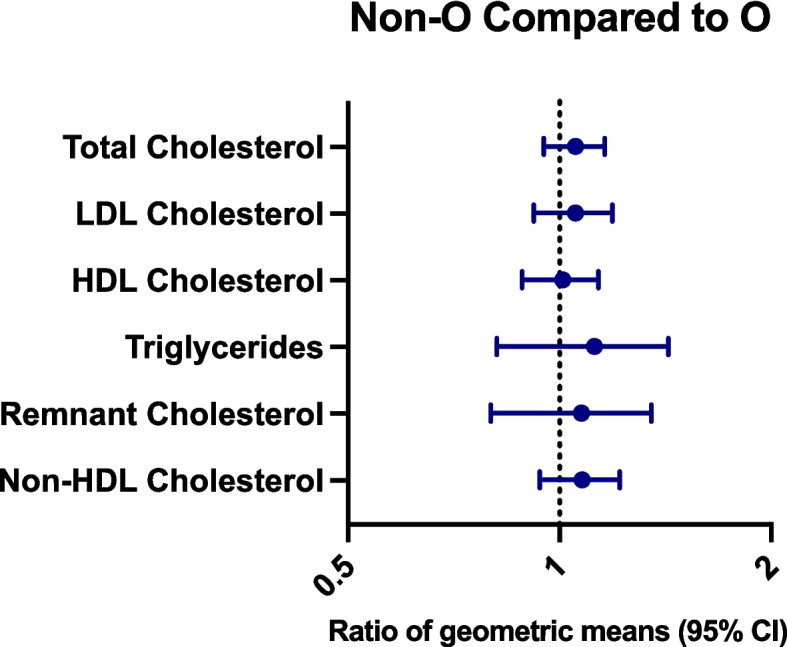
Differences in lipid levels in 40-year-old blood group non-O compared to O individuals

There were no statistically significant differences in lipid levels (assessed as total, LDL, HDL, remnant, non-HDL cholesterol and triglycerides) between the non-O and O blood groups. Neither in analyses stratified by sex nor in analyses stratified by both sex and age group (Supplementary Table 1). For RhD, male 40-year-old RhD** − **individuals had higher levels of non-HDL, LDL and remnant cholesterol compared to RhD + individuals, as seen in Fig. 2. This corresponds to a median non-HDL cholesterol level of 4.67 mmol/L in RhD** − **40y old male participants compared to 3.68 mmol/L in RhD + individuals, 3.70 mmol/L of LDL cholesterol in RhD** − **compared to 3.20 mmol/L in RhD + participants and 0.69 mmol/L of remnant cholesterol in RhD − compared to 0.53 mmol/L. However, as presented in Fig. 3, no differences were seen between the non-O blood group and O in this age group. Similarly, no differences in lipid levels depending on the RhD blood group were seen in the other male age groups or for women (supplementary Table 2).

In a subgroup analysis, individuals aged 50 and 60 with heredity for CVD (reported in a questionnaire when they were also 40 years old) were assessed. In this subgroup, we found no statistically significant differences in lipid levels depending on the ABO blood groups (supplementary Table 3) or the RhD blood groups (supplementary Table 4).

## Discussion

We found an association between dyslipidaemia and the RhD blood group. This was represented by 40-year-old male RhD − individuals with heredity for CVD, having increased levels of non-HDL cholesterol, LDL cholesterol, and remnant cholesterol compared to RhD + individuals. Corresponding associations were not seen among the male 50- and 60-year-old individuals (see graphical abstract). Thus, dyslipidaemia could be one of the mechanisms for our previous finding that the RhD blood group was associated with subclinical atherosclerosis in younger individuals [20]. No association was seen between dyslipidaemia and ABO blood groups.

Few studies are available that assess possible mechanisms behind the proposed susceptibility to CVD in different blood groups. This is partly due to the fact that there is still conflicting evidence regarding the association between ABO blood groups and atherosclerotic cardiovascular disease (ASCVD). For the RhD blood group, even less is known, as most previous studies investigating the association between CVD and blood groups have only considered the ABO system. Additionally, the literature is sparse on previously healthy individuals since most studies have examined high-risk patients with already known or suspected ASCVD and, consequently, probably advanced atherosclerotic lesions. Assessing underlying mechanisms in early atherosclerotic stages could shed light on the causes of cardiovascular disease, as this is still to be further elucidated to improve prevention strategies.

For the RhD blood group, the link to both health outcomes and possible underlying mechanisms is somewhat unknown. Recently there have been some large-scale studies assessing both ABO and RhD blood groups, one reported no association with CVD [5], and another found an association with increased risk for iliac aneurysm but no other ASCVD [6]. However, these are registry studies based upon reported ICD codes, with no plasma available for lipid analysis.

In the context of lipid parameter assessment, there are some important differences to consider in relation to the other studies. A previous study assessing lipid levels in 150 healthy Greek blood donors reported that RhD − presented a better lipid profile than RhD + [21]. This contrasts with our larger study, as we found RhD − individuals to have a more atherogenic lipid profile. Similar results were seen in another recent study, where RhD + subjects presented with lower HDL and higher LDL compared to RhD − subjects, but no differences were seen for ABO [28]. The study used lipid proteomics with nuclear magnetic resonance (NMR) for lipid analysis on 840 healthy Italian blood donors. However, both studies assessed blood donors, which contrasts with our results, as the sub-group of 40-year-olds in our study were included based on having heredity for CVD. In this age group, the RhD − male individuals presented with higher LDL, non-HDL, and remnant cholesterol levels. These studies also contrasts with the sub-groups aged 50 years old (included based on having one CVD risk factor) and those included by age 60 as the sole inclusion criterion, as we found no association with dyslipidaemia in these age groups. Lastly, in a retrospective study on 978 patients undergoing coronary artery bypass surgery due to multivessel CAD, RhD − individuals were reported to be associated with higher HDL cholesterol levels, but no other lipid parameters were associated [29]. A significant difference to our study, where there was no difference in HDL cholesterol, is that these patients had advanced atherosclerotic disease requiring surgery, while our participants are asymptomatic low to intermediate-risk individuals representing the pathophysiology in earlier stages of atherosclerosis. In addition, we excluded those with lipid-lowering therapy in our study, while the study on CAD reported no data on this.

This is the first study to assess derived lipid parameters such as non-HDL and remnant cholesterol in a population representative of early atherosclerotic disease in the context of blood groups. Derived lipid parameters, such as non-HDL cholesterol, are strongly associated with long-term risk for CVD [30] and are recommended in the current clinical guidelines for CVD risk assessment [31]. Calculated remnant cholesterol is also increasingly acknowledged for its role in atherosclerosis, as this considers the cholesterol content in triglyceride-rich remnants, which are also atherogenic and may explain part of the residual risk seen in ASCVD [32]. As there is evidence of discordance between non-HDL and remnant cholesterol and their disease susceptibility, it is essential to consider several lipid parameters to assess CVD risk and evaluate new risk markers [33]. For example, remnant cholesterol, but not LDL cholesterol, was shown to be associated with an increased risk for peripheral artery disease, while both elevated remnants and LDL explain the risk for myocardial infarction [34].

For the ABO blood group, some studies have stated that well-known risk factors for CVD, such as increased plasma lipids, are found in the non-O blood group as an explanation for their susceptibility to CVD, and a genom-wide association study (GWAS) study of Europeans also presented that among other genes, the ABO locus was associated with plasma lipids [35].

In most cases, the non-O blood groups have been associated with higher atherogenic plasma lipids levels, represented by increased total and/or LDL cholesterol [12, 13, 25]. Recently, non-O individuals were also found to be associated with downregulated proteins primarily associated with lipid metabolism [14]. By contrast, one study reported higher total cholesterol, LDL cholesterol, and triglycerides in blood group O [19]. Nevertheless, we found no association with dyslipidaemia, which is in line with a Chinese GWAS study assessing lipid-related loci associated with coronary artery disease, and found no association with ABO [15].

There are other aspects of the atherosclerotic process that can explain the discrepancy in these results. For example, non-O individuals have shown an increased CVD risk in epidemiologic studies [5, 6, 8, 10, 11, 36], but blood group O individuals have still been shown to have a higher atherosclerotic burden and a higher prevalence of hypercholesteremia, although the non-O individuals had a higher thrombus burden and larger myocardial infarcts [16]. The susceptibility to cardiovascular events in the non-O blood group can, therefore, also be attributed to other mechanisms in the atherosclerotic process besides dyslipidaemia, such as inflammatory pathways and coagulation pathways. This was previously presented in a study where non-O individuals were associated with increased levels of CRP, while no difference in lipid levels was found [15, 17]. Also, variations in sICAM and sP-selectin levels, which participate in the inflammatory processes by promoting the adhesion of leukocytes to the vascular endothelium, showed an association with the ABO locus [37, 38].

In the context of the coagulation pathway and concurrent thrombus formation, the ABO blood group has been recognised as a risk factor for venous thromboembolism for many years, where the non-O blood group confers a higher risk [8, 9]. This association has been attributed to lower plasma levels of von Willebrand factors and factor VIII in blood group O [39]. Consequently, this has also been a proposed mechanism for the susceptibility for CVD in blood group non-O. However, the evidence regarding atherosclerotic disease, especially for more stable forms such as CAD, is less clear [40]. The ABO blood group have been shown to impact platelet activation in the presence of already-existing coronary atherosclerosis [41]. Imaging studies in acute coronary syndrome patients have also seen that the incidence of atherosclerotic plaques does not differ between blood groups. Still, non-O individuals had more rupture-prone plaques [42]. Additionally, in a previous study in VIPVIZA, we found no association between the ABO blood groups and subclinical atherosclerosis assessed by ultrasonography [20]. Thus, the association of ABO and vWF with cardiovascular disease is mainly seen among those with an atherosclerotic event, including thrombosis, such as MI or ischemic stroke [14], whereas other factors influence the early and ongoing progression of atherosclerosis [43].

There are limitations of this study. One is the relatively small sample size in the age groups, which implies a loss of statistical power. Consequently, we could not evaluate the ABO blood groups separately. In this study, we compared non-O individuals with O individuals. Other more extensive studies have reported blood group A as the blood group associated with dyslipidaemia [13, 44]. We, therefore, conducted an A vs non-A analysis. However, we found no significant differences in lipid levels in analyses stratified by sex nor in analyses stratified by both sex and age group (supplementary Table 5). We also chose to present confidence intervals that were not adjusted to control the familywise error rate, as such adjustment would reduce statistical power. However, the choice of prioritising statistical power at the expense of increased risk of type 1-errors warrants caution in the interpretation of the findings.

Another limitation is that approximately 85% of all individuals in the VIPVIZA trial were registered in the SCANDAT register, with the possible risk of selection bias. Also, to avoid interference with lipid levels, individuals on lipid-lowering therapy and those with missing information about lipid-lowering treatment were excluded. Consequently, as with all exclusion criteria, there is a risk that the exclusion is not entirely random. In this study, the proportion of excluded individuals was larger in the 50 and 60-year-old groups, as treatment is more common with increased age.

There is a chance that those with dyslipidaemia in the older age groups might already have received statin treatment and, therefore, have already been excluded from this study. This might explain why we found no association between RhD − individuals and dyslipidaemia in the older age groups. Another explanation for the association only in 40-year-olds is that this age group is thoroughly selected for having heredity. As blood groups are hereditary, this may explaini part of the hereditary CVD risk. We therefore conducted a sub-analysis for 50- and 60-year-old individuals reporting heredity, but found no association with dyslipidemia. However, we think that this might be influenced by the fact that data for heredity in 50 and 60-year-olds are based on self-reported data from the questionnaire, which is in contrast to the 40-year-old group that had heredity as an inclusion criterion and therefore also were thoroughly interviewed regarding heredity. We argue that the age groups represent different populations, as the inclusion criteria in the study differ markedly, as an explanation to the different findings in different age groups.

A strength is that we assess asymptomatic individuals representing a low- to intermediate-risk population from a pragmatic trial [22]. The inclusion criteria for heredity in the 40-year-olds were thorough and based on a self-reported questionnaire and a follow-up health discussion with a trained nurse. Lastly, all blood samples were analysed at the same laboratory, avoiding interlaboratory variance.

Identifying and evaluating new markers associated with increased lipid levels, which are causal risk factors for CVD, might lead to improved prevention strategies by aiding in a more personalised risk stratification and adding information about non-traditional risk markers. Thus, the RhD blood group might be one of many future risk marker that will be combined for identifying individuals at increased CVD risk. This presents the clinical significance of blood groups beyond their use in transfusion medicine. However, further studies are needed to elucidate the role of the RhD blood group as a risk marker, which requires several criteria to be fulfilled previously described in detail [31]. This includes, e.g. assessment of the risk reclassification ability and the public health impact, which is not assessed in this descriptive study.

## Conclusions

We found an association between dyslipidaemia and the RhD blood group. This was represented by 40-year-old male RhD − individuals with heredity for CVD, having increased levels of non-HDL cholesterol, LDL cholesterol, and remnant cholesterol compared to RhD + individuals. No association was seen for the ABO blood group. This is one of the first studies to show associations between RhD and derived lipid parameters in asymptomatic individuals. Our findings primarily generate a further understanding of the atherosclerotic pathophysiology. Further elucidation is necessary to determine the role of blood groups as risk markers in clinical practice. However, since blood group antigens are constant inherited markers and one of the most readily available laboratory tests in clinical practice, they have the potential to be suitable risk markers in the future.

## Supplementary Information

Supplementary Material 1

## Data Availability

No datasets were generated or analysed during the current study.
