# Supplementary material for: ABO and RhD blood groups as contributors to dyslipidaemia – a cross-sectional study
Source: Lipids Health Dis. 2025 Jan 22;24:18. doi: 10.1186/s12944-025-02444-6 (PMC11753059; doi:10.1186/s12944-025-02444-6)
Supplement: Supplementary file 1 — Supplementary Material 1 [file 12944_2025_2444_MOESM1_ESM.docx]

Supplementary Table 1. The difference in lipid parameters in non-O compared to the O blood group.

| ABO | Men | | Women | |
| --- | --- | --- | --- | --- |
|  | GMR | CI 95% | GMR | CI 95% |
| Total cholesterol |  |  |  |  |
| All | 1.01 | 0.99; 1.04 | 1.00 | 0.98; 1.02 |
| 40 y | 1.05 | 0.95; 1.16 | 0.99 | 0.94; 1.05 |
| 50 y | 1.01 | 0.96;1.06 | 0.98 | 0.94; 1.01 |
| 60 y | 1.01 | 0.98; 1.04 | 1.01 | 0.99; 1.03 |
| LDL cholesterol |  |  |  |  |
| All | 1.02 | 0.98; 1.05 | 1.00 | 0.98; 1.03 |
| 40 y | 1.05 | 0.92; 1.19 | 1.01 | 0.93; 1.10 |
| 50 y | 1.00 | 0.93; 1.07 | 0.98 | 0.94; 1.03 |
| 60 y | 1.02 | 0.98; 1.06 | 1.01 | 0.97; 1.04 |
| HDL cholesterol |  |  |  |  |
| All | 1.01 | 0.98; 1.05 | 0.98 | 0.95; 1.01 |
| 40 y | 1.00 | 0.89; 1.14 | 0.96 | 0.88; 1.05 |
| 50 y | 1.04 | 0.98; 1.10 | 0.94 | 0.89; 0.99 |
| 60 y | 1.00 | 0.96; 1.04 | 1.00 | 0.97; 1.04 |
| Triglycerides |  |  |  |  |
| All | 1.02 | 0.96; 1.09 | 1.02 | 0.97; 1.07 |
| 40 y | 1.09 | 0.83; 1.44 | 1.00 | 0.87; 1.16 |
| 50 y | 1.03 | 0.90; 1.18 | 1.04 | 0.96; 1.13 |
| 60 y | 1.01 | 0.94; 1.09 | 1.00 | 0.95; 1.06 |
| Remnant cholesterol |  |  |  |  |
| All | 1.01 | 0.95; 1.07 | 1.02 | 0.98; 1.07 |
| 40 y | 1.05 | 0.81; 1.36 | 0.98 | 0.84; 1.14 |
| 50 y | 1.00 | 0.89; 1.12 | 1.05 | 0.97; 1.14 |
| 60 y | 1.01 | 0.94; 1.08 | 1.01 | 0.95; 1.07 |
| Non-HDL cholesterol |  |  |  |  |
| All | 1.02 | 0.98; 1.05 | 1.01 | 0.98; 1.04 |
| 40 y | 1.07 | 0.94; 1.22 | 1.01 | 0.93; 1.09 |
| 50 y | 0.99 | 0.94; 1.06 | 0.99 | 0.95; 1.04 |
| 60 y | 1.02 | 0.98; 1.06 | 1.01 | 0.98; 1.04 |
| GMR – geometric mean ratio (non-O/O). | | | | |

Supplementary Table 2. The difference in lipid parameters in RhD- compared to the RhD+ blood group

| RhD | Men | | Women | |
| --- | --- | --- | --- | --- |
|  | GMR | CI 95% | GMR | CI 95% |
| Total Cholesterol |  |  |  |  |
| All | 1.01 | 0.98; 1.05 | 1.00 | 0.97; 1.02 |
| 40 y | 1.13 | 1.00; 1.28 | 1.03 | 0.95; 1.11 |
| 50 y | 1.03 | 0.97;1.10 | 1.01 | 0.96; 1.05 |
| 60 y | 0.99 | 0.96; 1.03 | 0.99 | 0.96; 1.02 |
| LDL-Cholesterol |  |  |  |  |
| All | 1.02 | 0.97; 1.07 | 1.00 | 0.96; 1.04 |
| 40 y | **1.20** | **1.02; 1.41** | 1.03 | 0.91; 1.17 |
| 50 y | 1.05 | 0.95; 1.15 | 1.02 | 0.95; 1.09 |
| 60 y | 0.99 | 0.93; 1.04 | 0.98 | 0.94; 1.03 |
| HDL-Cholesterol |  |  |  |  |
| All | 0.97 | 0.93; 1.04 | 0.99 | 0.95; 1.03 |
| 40 y | 0.90 | 0.76; 1.05 | 1.09 | 0.96; 1.23 |
| 50 y | 0.97 | 0.89; 1.05 | 1.00 | 0.93; 1.07 |
| 60 y | 0.98 | 0.93; 1.04 | 0.97 | 0.92; 1.02 |
| Triglycerides |  |  |  |  |
| All | 1.09 | 1.00; 1.19 | 1.01 | 0.95; 1.08 |
| 40 y | 1.21 | 0.85; 1.72 | 0.85 | 0.69; 1.05 |
| 50 y | 1.11 | 0.92; 1.34 | 0.98 | 0.87; 1.11 |
| 60 y | 1.07 | 0.97; 1.19 | 1.05 | 0.97; 1.13 |
| Remnant Cholesterol |  |  |  |  |
| All | 1.08 | 0.99; 1.17 | 1.00 | 0.93; 1.06 |
| 40 y | **1.38** | **1.00; 1.92** | 0.84 | 0.68; 1.04 |
| 50 y | 1.01 | 0.86; 1.19 | 0.96 | 0.85; 1.08 |
| 60 y | 1.05 | 0.98; 1.19 | 1.04 | 0.96; 1.12 |
| Non-HDL Cholesterol |  |  |  |  |
| All | 1.03 | 0.98; 1.07 | 1.00 | 0.96; 1.04 |
| 40 y | **1.21** | **1.03; 1.43** | 1.00 | 0.89; 1.12 |
| 50 y | 1.05 | 0.97; 1.15 | 1.01 | 0.95; 1.07 |
| 60 y | 1.00 | 0.95; 1.05 | 0.99 | 0.95; 1.04 |
| GMR – geometric mean ratio (RhD-/RhD+). Statistically significant values presented in bold. | | | | |

Supplementary Table 3. The difference in lipid parameters in non-O compared to the O blood group in individuals reporting heredity for CVD

| ABO | Men | | Women | |
| --- | --- | --- | --- | --- |
|  | GMR | CI 95% | GMR | CI 95% |
| Total Cholesterol |  |  |  |  |
| 50 y | 0.96 | 0.83; 1.11 | 0.97 | 0.91; 1.04 |
| 60 y | 1.04 | 0.96; 1.13 | 1.02 | 0.95; 1.10 |
| LDL-Cholesterol |  |  |  |  |
| 50 y | 0.91 | 0.74; 1.13 | 0.99 | 0.89; 1.11 |
| 60 y | 1.06 | 0.94; 1.19 | 1.03 | 0.93; 1.14 |
| HDL-Cholesterol |  |  |  |  |
| 50 y | 0.99 | 0.87; 1.13 | 0.92 | 0.82; 1.03 |
| 60 y | 0.99 | 0.88; 1.12 | 1.05 | 0.95; 1.17 |
| Triglycerides |  |  |  |  |
| 50 y | 1.21 | 0.93; 1.58 | 0.98 | 0.82; 1.18 |
| 60 y | 1.03 | 0.81; 1.30 | 0.94 | 0.80; 1.10 |
| Remnant Cholesterol |  |  |  |  |
| 50 y | 1.20 | 0.96; 1.51 | 1.01 | 0.85; 1.21 |
| 60 y | 1.12 | 0.90; 1.39 | 0.94 | 0.79; 1.11 |
| Non-HDL Cholesterol |  |  |  |  |
| 50 y | 0.95 | 0.78; 1.14 | 1.00 | 0.90; 1.11 |
| 60 y | 1.06 | 0.95; 1.18 | 1.02 | 0.93; 1.12 |
| GMR – geometric mean ratio (non-O/O). | | | | |

Supplementary Table 4. The difference in lipid parameters in RhD- compared to the RhD+ blood group in individuals reporting heredity for CVD

| RhD | Men | | Women | |
| --- | --- | --- | --- | --- |
|  | GMR | CI 95% | GMR | CI 95% |
| Total Cholesterol |  |  |  |  |
| 50 y | 1.00 | 0.82; 1.21 | 0.98 | 0.88; 1.09 |
| 60 y | 1.05 | 0.93; 1.17 | 0.95 | 0.85; 1.07 |
| LDL-Cholesterol |  |  |  |  |
| 50 y | 0.97 | 0.74; 1.29 | 0.93 | 0.78; 1.11 |
| 60 y | 1.07 | 0.91; 1.27 | 0.98 | 0.84; 1.14 |
| HDL-Cholesterol |  |  |  |  |
| 50 y | 1.01 | 0.85; 1.20 | 1.12 | 0.93; 1.34 |
| 60 y | 0.94 | 0.79; 1.11 | 0.89 | 0.76; 1.05 |
| Triglycerides |  |  |  |  |
| 50 y | 1.05 | 0.74; 1.49 | 0.80 | 0.60; 1.08 |
| 60 y | 1.17 | 0.84; 1.63 | 1.04 | 0.81; 1.34 |
| Remnant Cholesterol |  |  |  |  |
| 50 y | 0.93 | 0.69; 1.27 | 0.78 | 0.59; 1.04 |
| 60 y | 1.17 | 0.86; 1.59 | 1.02 | 0.79; 1.32 |
| Non-HDL Cholesterol |  |  |  |  |
| 50 y | 1.01 | 0.79; 1.29 | 0.91 | 0.77; 1.07 |
| 60 y | 1.08 | 0.93; 1.26 | 0.98 | 0.85; 1.14 |
| GMR – geometric mean ratio (RhD-/RhD) | | | | |

| ABO | Men | | Women | |
| --- | --- | --- | --- | --- |
|  | GMR | CI 95% | GMR | CI 95% |
| Total cholesterol |  |  |  |  |
| All | 0.98 | 0.96; 1.00 | 0.99 | 0.97; 1.00 |
| 40 y | 0.93 | 0.84; 1.02 | 0.99 | 0.94; 1.05 |
| 50 y | 0.99 | 0.94; 1.03 | 1.00 | 0.97; 1.04 |
| 60 y | 0.98 | 0.95; 1.01 | 0.98 | 0.96; 1.00 |
| LDL cholesterol |  |  |  |  |
| All | 0.97 | 0.93; 1.00 | 0.98 | 0.95; 1.00 |
| 40 y | 0.93 | 0.82; 1.06 | 0.97 | 0.89; 1.06 |
| 50 y | 0.97 | 0.91; 1.04 | 1.01 | 0.96; 1.06 |
| 60 y | 0.97 | 0.93; 1.01 | 0.97 | 0.93; 1.00 |
| HDL cholesterol |  |  |  |  |
| All | 0.99 | 0.96; 1.02 | 1.00 | 0.98; 1.03 |
| 40 y | 1.00 | 0.88; 1.13 | 1.03 | 0.95; 1.13 |
| 50 y | 0.95 | 0.90; 1.01 | 1.03 | 0.98; 1.08 |
| 60 y | 1.00 | 0.96; 1.04 | 0.99 | 0.95; 1.02 |
| Triglycerides |  |  |  |  |
| All | 0.99 | 0.93; 1.06 | 0.98 | 0.94; 1.03 |
| 40 y | 0.83 | 0.64; 1.08 | 1.04 | 0.89; 1.20 |
| 50 y | 1.03 | 0.90; 1.17 | 0.94 | 0.87; 1.02 |
| 60 y | 0.99 | 0.92; 1.07 | 1.00 | 0.94; 1.06 |
| Remnant cholesterol |  |  |  |  |
| All | 1.00 | 0.94; 1.06 | 0.97 | 0.93; 1.02 |
| 40 y | 0.87 | 0.67; 1.12 | 1.07 | 0.91; 1.24 |
| 50 y | 1.04 | 0.93; 1.16 | 0.93 | 0.86; 1.01 |
| 60 y | 0.99 | 0.92; 1.07 | 0.99 | 0.94; 1.05 |
| Non-HDL cholesterol |  |  |  |  |
| All | 0.97 | 0.94; 1.00 | 0.98 | 0.95; 1.00 |
| 40 y | 0.91 | 0.80; 1.03 | 0.98 | 0.90; 1.07 |
| 50 y | 1.00 | 0.94; 1.06 | 0.99 | 0.95; 1.04 |
| 60 y | 0.97 | 0.93; 1.01 | 0.97 | 0.94; 1.00 |
| GMR – geometric mean ratio (non-A/A). | | | | |

Supplementary Table 5. The difference in lipid parameters in non-A compared to the A blood group.
